# Evaluation of Dimensional Changes According to Aging Period and Postcuring Time of 3D-Printed Denture Base Prostheses: An In Vitro Study

**DOI:** 10.3390/ma14206185

**Published:** 2021-10-18

**Authors:** Seung-Ho Shin, Re-Mee Doh, Jung-Hwa Lim, Jae-Sung Kwon, June-Sung Shim, Jong-Eun Kim

**Affiliations:** 1 BK21 FOUR Project, Oral Research Science Center, Department of Prosthodontics, Yonsei University College of Dentistry, Yonsei-ro 50-1, Seoul 03722, Korea; shin506@prostholabs.com (S.-H.S.); erin850313@gmail.com (J.-H.L.); 2 Department of Advanced General Dentistry, College of Dentistry, Dankook University, Cheonan 31116, Korea; remeedoh@dankook.ac.kr; 3 Department and Research Institute of Dental Biomaterials and Bioengineering, Yonsei University College of Dentistry, Seoul 03722, Korea; jkwon@yuhs.ac; 4 Department of Prosthodontics, Yonsei University College of Dentistry, Yonsei-ro 50-1, Seoul 03722, Korea; jfshim@yuhs.ac

**Keywords:** 3D printing, additive manufacturing, aging, trueness, postcuring, dimensional change

## Abstract

During the three-dimensional (3D) printing process of a dental prosthesis, using photopolymer resin, partially polymerized resin is further cured through the postcuring process that proceeds after the printing, which improves the stability of the printed product. The mechanical properties of the end product are known to be poor if the postcuring time is insufficient. Therefore, this study evaluated the effect of the postcuring time of the 3D-printed denture base on its dimensional stability, according to the aging period. The 3D prints were processed after designing maxillary and mandibular denture bases, and after the following postcuring times were applied: no postcuring, and 5, 15, 30, and 60 min. The dimensional stability change of the denture base was evaluated and analyzed for 28 days after the postcuring process. The trueness analysis indicated that the mandibular denture base had lower output accuracy than the maxillary denture base, and the dimensional stability change increased as postcuring progressed. In the no postcuring group for the mandible, the error value was 201.1 ± 5.5 µm (mean ± standard deviation) after 28 days, whereas it was 125.7 ± 13.0 µm in the 60 min postcuring group. For both the maxilla and the mandible, shorter postcuring times induced larger dimensional stability changes during the aging process. These findings indicate that in order to manufacture a denture base with dimensional stability, a sufficient postcuring process is required during the processing stage.

## 1. Introduction

Recently, digital dentistry development, represented by computer-aided design/computer aided manufacturing (CAD/CAM), has significantly progressed in digital dental procedures of the oral cavity [1], creating more general access to digital oral information for patients [2]. This digitized oral information of the patient can be also stored, retrieved, transmitted, and analyzed within the software, and the dental CAD program can help dentists and technicians to construct dental design prostheses faster [3,4]. CAD/CAM developments have resulted in many changes in the processing of dental prostheses [5]. Dental prosthesis manufacturing using the subtractive manufacturing (SM) method constructs dental prostheses by processing existing blocks, such as those made from polymethylmethacrylate and zirconia. This is the most widely used technology and is faster and simpler than conventional dental prosthesis production using casting. However, it has disadvantages, such as increased cutting force due to wear on the milling tool, restrictions in reproducibility according to tool size, difficulty in manufacturing complex shapes, and producing large amounts of unused and discarded material [6]. However, three-dimensional (3D) printing, additive manufacturing, or rapid prototyping methods compensate for the disadvantages of the abovementioned SM methods [7]. These manufacturing methods have the great advantage of the hue and composition allowing the selection of other suitable polymers for the production of various prostheses, such as in the production of computed tomography stents, provisional crowns, record bases, and dentures [8]. As 3D printing materials, such as ceramics and metals, have recently become more diverse, various applications in the dental field have also become available [9]. The manufacturing time and material consumption of the dental prosthesis can also be greatly reduced, and it also has a manufacturing reproducibility [3,10].

Additive manufacturing, represented by 3D printing, uses photopolymer resin and produces output by polymerization, while stacking layers [11]. This method has considerations that did not need to be addressed during the conventional manufacturing process of prostheses [12]. For porous materials, such as individual trays or temporary crowns, a more accurate manufacturing process is also important, but a faster manufacturing process is often required. On the other hand, when used as a final prosthesis, aspects such as precision, strength, and dimensional stability are important [13]. Prostheses with a large dimensional and contact area with the oral cavity, such as a denture base, also primarily require long-term dimensional stability of the denture base and need precisely reproduced occlusion with the oral cavity [14,15]. Errors accumulate during the 3D printing process of the prosthesis, and the patient may have an increased chair time to correct the error; if the error cannot be corrected, the prosthesis needs to be manufactured from the beginning again [13,16]. The accuracy and mechanical properties of 3D printing can be affected by the parameters adjusted during the 3D printing process, such as the light intensity, output position, build angle, thickness and number of layers, and configuration of the printing support structure. The heterogeneity of these combinations has also been reported [11,15,17,18,19,20,21,22]. The postcuring process is also very important when manufacturing a dental prosthesis with 3D printing and can affect its overall quality, such as its physical strength and biological properties. Many previous studies reported that increased postcuring time and high-temperature postcuring makes the mechanical properties, such as the fracture strength and surface hardness, and biologically stable results, such as cytotoxicity, and cell viability, greatly improved [23]. Another study found that the degree of conversion of 3D-printed resin and the fracture strength of a three unit bridge vary according to the type of postcuring equipment and the peak wavelength of the postcuring light [24,25,26].

Another previous study investigated the dimensional deformation of the 3D printing model, according to its aging period [27]. The model in that study involved the same postcuring times being applied after 3D printing. To the authors’ knowledge, no research results have been reported on the effect of the different postcuring times applied after 3D printing on the long-term dimensional stability, according to its aging period. Therefore, in the present study, we paid attention to the fact that the photopolymer resin needs sufficient postcuring after printing to become stable, and we considered that the postcuring time will affect the dimensional stability of the completed prosthesis due to the characteristics of the material. The purpose of this study was therefore to determine the effect of the postcuring time of 3D-printed denture base prostheses on their long term dimensional stability, according to the aging period. The null hypotheses of this study were as follows: (1) there is no difference in the dimensional stability of the maxillary and mandibular arches in the 3D-printed denture base, and (2) the postcuring time of denture bases does not affect their long-term dimensional stability.

## 2. Materials and Methods

**Figure 1:**
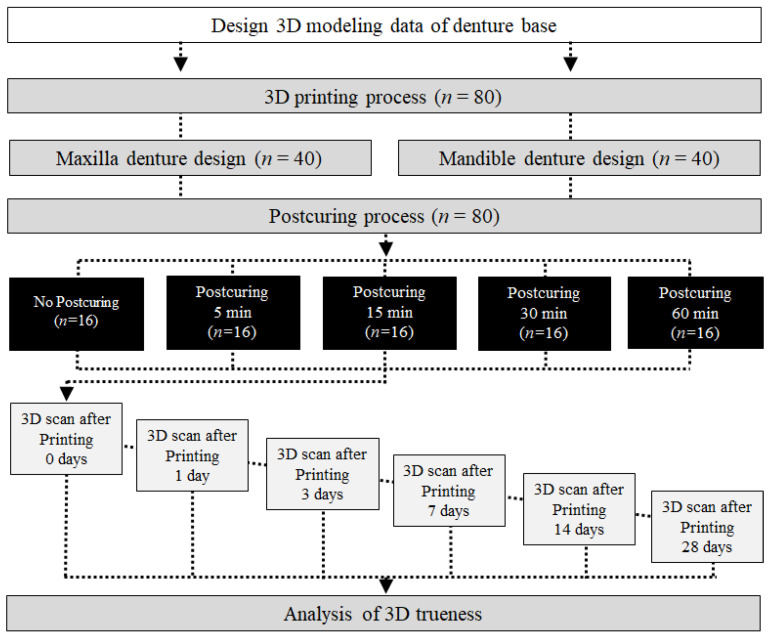
Flowchart of the study design. *n* = number of specimens.

Figure 1 illustrates the model design and overall experimental workflow of this study.

**Figure 2:**
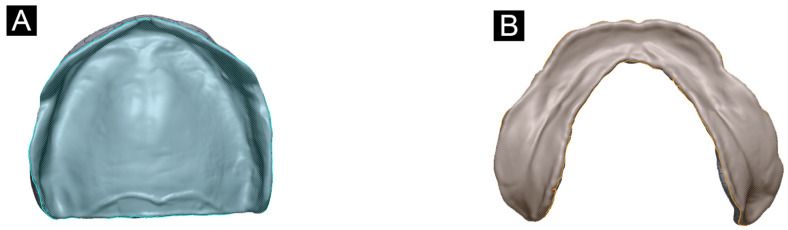
Design of the maxillary (**A**) and mandibular (**B**) denture bases. The inner area of the denture base was selected to evaluate accuracy.

To design a denture base, reference model selection was performed using the following process: according to the classification system from the American College of Prosthodontists, an edentulous maxillary and mandibular cast with class I, type A, residual ridge morphology and no severe undercuts was selected as the reference. The maxillary and mandibular models were scanned, using a tabletop scanner that has <7 microns accuracy to ISO 12836 (Identica T500, Medit, Seoul, Korea) and then exported as a digital file in the Standard Triangulated Language (STL) format. The converted design file was imported into modeling software (Meshmixer, Autodesk, San Rafael, CA, USA), and the maxilla and mandible denture bases were designed based on this edentulous model (Figure 2).

For the design of each printed denture base, photopolymer resin (MAZIC^®^ D model, Vericom, Chuncheon, Korea) and an LCD type 3D printer (Phrozen Shuffle, Phrozen, Hsinchu, Taiwan) were used. The components of photopolymer resin and specifications of this 3D printer are given in Table 1 and Table 2.

The designed STL format file was imported into the Nano DLP open-source slicing software (Nano DLP, Richmond Hill, ON, Canada), which was used to form a support structure to form and establish the 3D printing parameters. Before creating the support structure, the denture base was rotated 120 degrees from its forward direction to minimize contact with the gingival palatal part with support structure, because the palatal part was used for the 3D analysis. Each printing layer had a thickness of 100 μm and was cured for 15 s. The support structure was attached to the lingual side of the denture base without contacting the gingiva. For the support structure, a separate slicing program (Chitu DLP slicer Version 1.3.6, Shenzhen, China) was used, and the automatic function was used with a support density of 20%. After support alignment, manual support rearrangement was performed because of stable printing. The minimum height of the support structure is 5 mm, the attachment angle is 45 degrees, and the thickness of the support structure is 1 mm. This support structure was maintained during the postcuring progress and was removed after its completion. All denture bases were washed at 20 °C and 50% humidity and 90% isopropyl alcohol, using a washing machine (Formwasher, Formlabs, Somerville, MA, USA), and then each group underwent postcuring, using a UV polymerization unit (MP100, Hepsiba Corporation, Incheon, Korea). Polymerization was performed for 5 min, 15 min, 30 min, or 60 min, with the no-curing group was a control group, not postcured. The postcuring device covered the spectral range of 365–420 nm, using a 74 W lamp. All denture bases were stored at 20 °C and 50% humidity without direct sunlight exposure after printing. Each denture base was scanned once after the printing and postcuring process with a tabletop 3D scanner in the order of the printing day: 1st, 3rd, 7th, 14th, and 28th. It is assumed that each post-curing time and aging date will vary in the storage environment after printing, and the setting of the postcuring time refers to a past study [26] in which the strength increases as the postcuring time increases, and the aging date refers to the aging study of up to 4 weeks after printing the model in a past study [27]. The scanned data were saved in the STL format and applied to a 3D analysis software program (Geomagic Control X, 3D Systems, Rock Hill, SC, USA).

The scan data of each group were superimposed on the outline of the denture base design reference data, and trueness was calculated after complete alignment. A best-fit process was performed during the alignment process of scan data for trueness. The best-fit process calculated and registered how the mesh point of the loaded 3D model deviated from the reference model and evaluated the deviation of the mesh set based on the calculated deviation of each point and its dependence on the variable. A sorting process was used to find the optimal sort. After the maxillary and mandibular prostheses were included in the scan data corresponding to the denture base reference data and storage time of the inner part of the denture base that contacted the gingiva, the trueness values were compared by calculating surface-deviation values, using the root-mean-square error (RMSE). RMSE is a mean calculated as the square root of the mean square by which an error index can be identified in units similar to the actual value and is applied to measure the dimensional and shape variability of a surface [28]. This study, therefore, determined RMSE as error values of trueness, as follows:

(1) $$\mathrm{RMSE}\;=\frac{1}{\sqrt{n}}\cdot \sqrt{\overset{n}{\underset{i=1}{\sum }}{\left(x_{1,i}-x_{2,i}\right)}^{2}}$$

where $x_{1}$ and $x_{2}\;$ represent the predicted and actual values, respectively, and *i* refers to the order of the starting terms to determine the sum of the formula; since it starts from the first term, *i* is fixed at 1. Statistical analysis was performed by calculating the RMSE values according to the above formula. An RMSE farther from zero indicates larger dimensional errors relative to the reference model, and vice versa.

All scan data used to calculate the RMSE values were statistically analyzed to determine the trueness values of each group, and the average discrepancy value obtained from comparing surface RMSE data set was used for the statistical analysis. All statistical analyses were performed using SPSS statistical software (IBM SPSS Statistics for Windows, Version 25.0, IBM Corp, Armonk, NY, USA). Levene’s test was applied to all acquired data to evaluate the homoscedasticity, and the Shapiro–Wilk test was used to evaluate normality. A three-way ANOVA test was performed to identify differences between groups according to the arch position, postcuring time, and elapsed time after printing, and one-way ANOVA was used to compare the dimensional changes, according to the postcuring time within the same aging-period group, and the elapsed time after printing. The Bonferroni correction was used as a post hoc test (*p* = 0.05).

## 3. Results

**Figure 3:**
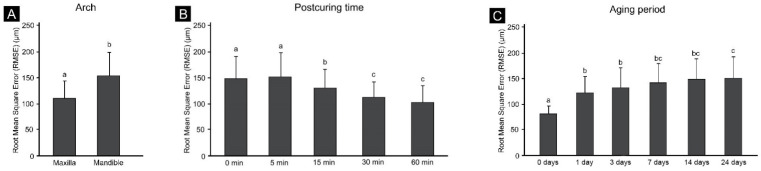
(**A**) Dimensional difference according to arch position, (**B**) dimensional difference after postcuring; and (**C**) dimensional difference, according to model aging period. Different lower-case letters indicate significant differences (*p* < 0.05).

The groups without postcuring and with 5 min of postcuring had the largest errors (Figure 3B). In the error graph for each elapsed period after printing, the error appeared to gradually increase from day 0. As the aging period increased, the dimensional change gradually decreased (Figure 3C).

**Figure 4:**
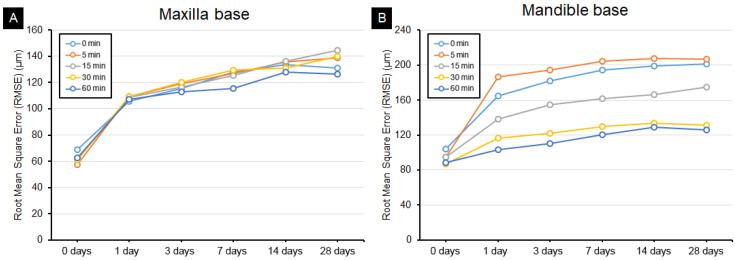
Trueness according to aging period after the printing process in the (**A**) maxilla base and (**B**) mandible base.

According to the overall trueness result (Table 3), the RMSE value comparisons revealed the trueness of the maxillary (Figure 4A) and mandibular (Figure 4B) denture bases, and large shape deformations were identified between days 0 and 1 in both the mandible and maxilla. The maxillary denture base had similar dimensional stabilities regardless of the postcuring time. The mandibular denture base had a large RMSE value over time in the groups with short postcuring times, indicating a low dimensional stability. Overall, the maxillary denture base had a lower RMSE value than the mandibular denture base, indicating a higher dimensional stability. The RMSE value for the mandibular denture base was lowest in the 60 min postcuring group and highest in the no-postcuring and 5 min postcuring groups

The three-way ANOVA indicated that dimensional stability was significantly affected by the postcuring time dimensional stability (F = 51.07, *p* < 0.001), the elapsed time after printing (F = 123.11, *p* < 0.001), and the arch position (F = 280.15, *p* < 0.001). The postcuring time and arch position also had a mutually significant effect (F = 43.40, *p* < 0.001), as did the elapsed time after printing and the postcuring time (F = 1.73, *p* = 0.027). However, the elapsed time after printing and arch position did not significantly affect each other (F = 99.33, *p* = 0.962). The elapsed time after printing, postcuring time, and denture base shape also did not significantly affect each other (F = 1.32, *p* = 0.164). The results of the three-way ANOVA are presented in Figure 3. The maxilla was significantly more accurate than the mandible (F = 280.15, *p* < 0.001) (Figure 3A), and it had a smaller dimensional stability change and an increased dimensional stability as the postcuring time increased.

**Figure 5:**
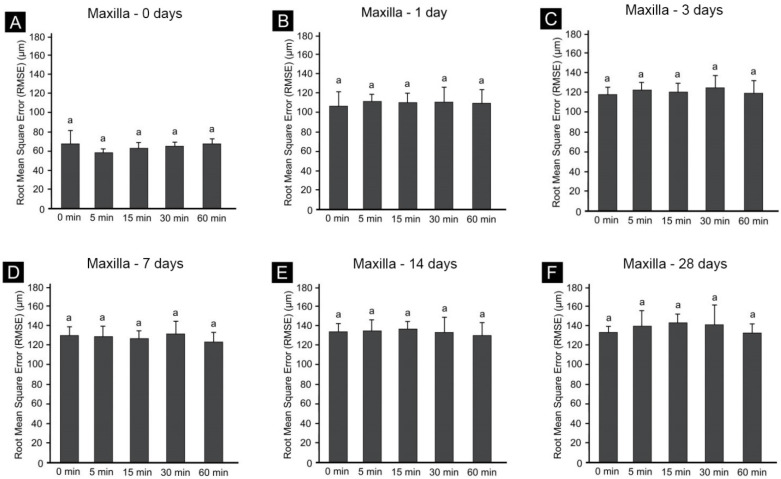
RMSE values at (**A**) 0 days, (**B**) 1 day, (**C**) 3 days, (**D**) 7 days, (**E**) 14 days and (**F**) 28 days after the maxilla printing process. Data are mean and standard deviation values. Different lowercase letters in a single graph indicate significant differences.

**Figure 6:**
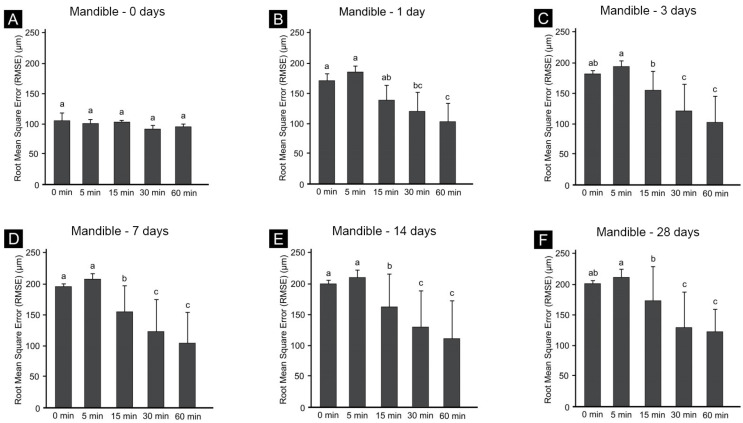
RMSE values at (**A**) 0 days, (**B**) 1 day, (**C**) 3 days, (**D**) 7 days, (**E**) 14 days and (**F**) 28 days after the mandible printing process. Data are mean and standard-deviation values. Different lowercase letters in the same graph indicate significant differences.

Graphs of the individual aging periods in the groups for the maxilla (Figure 5) and mandible (Figure 6) revealed shape changes according to the postcuring time for the same aging period (Figure 5A,B and Figure 6A,B). However, the dimensional changes in the maxilla at the same postcuring time was not significantly affected by the postcuring time, and all of them were similar (Figure 5). However, there was a significant dimensional change in the mandible, according to the postcuring time within the same aging period: as the postcuring time increased, the dimensional stability increased significantly (Figure 6).

**Figure 7:**
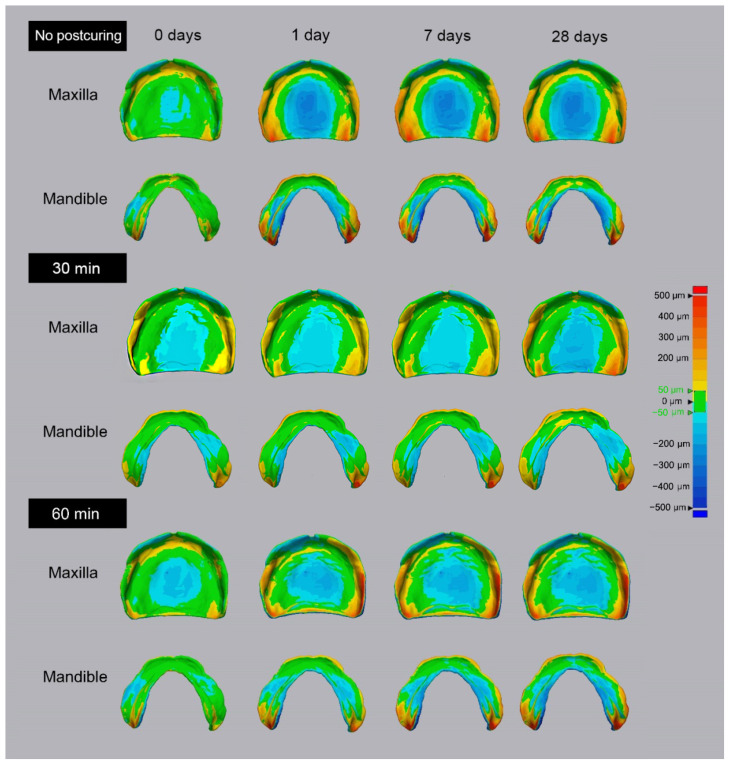
The 3D color maps at 0 days, 1 day, 7 days and 28 days after printing of the no postcuring, and 30 min and 60 min postcuring groups. Comparing the no postcuring group with the 30 min and 60 min postcuring groups, postcuring time increases, and the dimensional change is decreasing.

The 3D color map analysis revealed difference in dimensional deformations between the no-postcuring and 60 min postcuring groups (Figure 7). The no-postcuring and 60 min postcuring groups exhibited the most significant dimensional changes, strengthening conclusions regarding their dimensional changes. In both the maxillary and mandibular color maps, the dimensional deformations between days 0 and 1 were remarkable. In the maxilla, lingual contraction of the palatal region and shape deformation around the maxillary tuberosity and hamular notch were measurable from day 1 in both the no-postcuring and 60 min postcuring groups. However, the deformation dimensional was likely to be greater in the 60 min postcuring group than in the no postcuring group. The mandible appeared to be distorted at the mylohyoid muscle and retromolar pad on the lingual side of the posterior region. The mandible also had greater dimensional deformation in the no-postcuring group than in the 60 min postcuring group.

## 4. Discussion

This study evaluated dimensional changes, according to the postcuring times and elapsed times after printing in order to determine the optimal postcuring time for minimizing the dimensional changes in 3D printed maxillary and mandibular denture bases. For every group, a post hoc power revealed that analysis using G*power estimate respondent sample size of 8 in each group had a 99% power to detect the difference in dimensional deformations between the reference design and the 3D scan file with the highest effect. In this study, the thickness of the printing layer was 100 μm. A past study [20] confirmed that there was no statistical difference, according to the thickness of the printing layer, so this study suggested printing at 100 μm/layer. Various past studies [29,30] also used 100 μm/layer printing for their dimensional stability evaluation. In the previous study [18,31,32], it was confirmed that less than 100 µm is a clinically applicable error range. The sample size for the dimensional deformations evaluation was sufficient. The results indicated that the mandible had a larger dimensional change than the maxillary when postcuring was performed after 3D printing. Dimensional changes according to aging periods were small when the postcuring time was insufficient. Therefore, the first null hypothesis that there would be no difference in the dimensional stability between the maxillary and mandibular arches was rejected, as was the second null hypothesis that differences in postcuring times for the denture base would not affect the long-term dimensional stability.

This study indicated that dimensional changes occur continuously when the prosthesis is manufactured using 3D printing technology with photopolymer resin. Joda et al. [27] printed full-arch models with a Digital Light Processing type 3D printer and then performed postcuring and aging from 1 to 4 weeks on 10 tooth models stored at a constant 20 °C and 50% humidity. The resulting dimensional deformations were analyzed, which revealed that 1 week after printing, the storage group had an average dimensional deformation of 4.0 µm, the 3 week group had an average error of 6.4 µm, and the 4 week group had an average error of 8.9 µm, and that a statistically significant change occurred after 3 weeks. The dimensional change in that study occurred over the passage of time, even after 3D printing and the postcuring process, which was similar to the dimensional change pattern over time found in this study. Therefore, if a 3D-printed model is used to produce prostheses, it is suggested that it should be used as soon as possible, and definitely for no longer than 3–4 weeks [27] However, the present study indicated that the dimensional change after 28 days exceeded the minimum value of 100 µm and the maximum value of 200 µm, which was thought to have occurred due to the denture base being used as the evaluation design in our study. Unlike the dental model, the denture bases are generally thin, and therefore, have a weaker resistance to shrinkage and distortion, compared to a model [33,34]. This indicates that the dimensional error of the denture base was larger over time than the long-term dimensional stability data reported by Joda et al., who investigated a model.

In the trueness analysis, according to postcuring times and aging periods, dimensional changes in the maxilla and mandible appeared to be the main cause of shrinkage during the polymerization process [35,36]. Comparing the maxillary and mandibular arches revealed that the dimensional deformation of the mandibular denture base was larger than that of the maxillary denture base. The three-way ANOVA results indicated that the maxillary and mandibular denture bases had dimensional errors of 113.4 µm and 147.6 µm, respectively. In addition, both the maxilla and the mandible exhibited their largest changes during the first day. The maxilla had very similar changes regardless of the postcuring time, whereas the mandible had markedly different dimensional changes with different postcuring times. Shin et al. [37] evaluated the accuracy of a 3D-printed dental model and reported that the design filled with the palatal surface was more accurate than the tooth model of the full arch design without the palatal surface. The structure of the plate was reported to increase the stability of the 3D-printed dental model. In that study, when designing the maxillary and mandibular denture bases, the palatal part of the maxilla played a role in the cross-arch stabilization, and it is therefore thought that it shrunk less than the mandible because it was resistant to overall deformation in the shrinkage process caused by the postcuring process. The postcuring process induces additional polymerization in the remaining uncured part; mixing the uncured part with the hardened part before the postcuring process was reported to affect its dimensional stability [38]. Shrinkage during postcuring was also reported to affect the accuracy of 3D-printed objects [39,40,41,42].

In the present study, the dimensional change immediately after the postcuring process did not differ significantly with the postcuring time in both the maxillary and mandibular arches (*p* > 0.05). This was inconsistent with the results reported by McCarty et al. [43], who evaluated the postcuring time and dimensional deformation of a clear aligner printed with an SLA-type 3D printer. After the 3D printing of the clear aligner, postcuring was performed at a temperature of 80 °C for 20 min and 40 min, and the dimensional change was compared. The 20 min and 40 min postcuring groups had errors of 0.163 ± 0.017 mm and 0.148 ± 0.006 mm (mean ± standard deviation), respectively. They reported that the 40 min postcuring group was more accurate than the 20 min postcuring group. Regarding the results of the present study, the long term dimensional stability increased with the postcuring time. For the maxillary denture base, the postcuring time did not significantly affect the dimensional stability, but the mandibular denture base did show significant differences in the dimensional stabilities. For the mandibular denture base, the no postcuring and the 5 min and 15 min postcuring groups had larger dimensional changes as the aging period increased. However, in the 30 min postcuring group, there was very little dimensional change, according to increases in the aging period. The postcuring time of up to 15 min did not seem to sufficiently increase the polymerization rate of the unpolymerized part of the 3D-printed denture base, and the residual stress due to anisotropy of the base seemed to have induced the dimensional change. A sufficient postcuring time of at least 30 min may contribute to maintaining the dimensional stability of 3D-printed denture bases by improving the polymerization rate and anisotropic resolution.

Jindal et al. [26] indicated that the strength increases alongside the postcuring time when 3D printing with the photopolymer resin. Kim et al. [23] reported that the mechanical properties deteriorated and biological stability was low when the degree of resin polymerization was insufficient. These different results can be attributed to the polymerization rate; the Fourier-transform infrared spectroscopy analysis indicated that longer postcuring times resulted in better polymerization rates. That previous study recommended a postcuring time of at least 60 min [23]. In general, printing with photopolymer resin induces a polymerization rate that can maintain printed shapes immediately after printing, but postcuring is still essential since they are not completely polymerized. When printing using photopolymer resin, it was reported that less curing occurs closer to the edge of each layer [38]. Therefore, it is considered necessary to sufficiently harden the outer surface of the output by postcuring to suppress dimensional deformation as much as possible.

The accuracy of the denture base contributes to retention by improving the fit of the denture to the oral cavity, and it plays a role in improving the masticatory function of the patient using the denture and helps psychological stability. In addition, using the denture base as a record base for edentulous patients can affect the accuracy of jaw relation and the occlusion accuracy of the final denture. When used as a customized tray of the same type, the denture base can affect the dimensional stability after the impression is taken and can therefore be a vital important factor for determining the quality of the dentures. Due to recent developments in digital dentistry, 3D-printing technology has increasingly been used for dental prostheses manufacturing and is replacing the traditional method in many fields. This study indicated that a sufficient postcuring time for the 3D-printed denture base plays a crucial role in reducing long-term dimensional changes, and it is, therefore, recommended to provide a sufficient postcuring time of at least 30 min after the 3D-printing process. In particular, since dimensional changes occur mostly within 1 day after 3D printing, proceeding with the postcuring process immediately after 3D printing will be helpful. Controlling the overall 3D printing process is very important to improving its accuracy and as indicated in this study, providing sufficient postcuring time is also one of the most important factors.

Since this study focused on the effect of postcuring time on long-term dimensional stability, single types of 3D printing equipment, postcuring equipment, and photopolymer resin were used. However, the output method of the 3D printer equipment, the light source of the postcuring equipment, and the type of 3D-printed resin may result in differences in the polymerization rate and dimensional stability immediately after printing or after postcuring. Therefore, examining the results obtained when using various equipment and materials will improve the overall understanding and provide insight, and so this is considered an important future research direction. In addition, if we evaluate both the design of the denture base and how the postcuring time affects long-term dimensional stability in designs with various thicknesses and shapes, the use and understanding of 3D-printing technology to fabricate prostheses will be improved for dentists in clinical settings.

In the case of denture prostheses, dimensional changes are known to be prevented when storing the prostheses under water immediately after production is completed [44]. In addition, in the case of 3D-printed dentures, most processes, including the production process, and various parts, including materials, are different from traditional dentures [45]. However, it was confirmed that digitally manufactured dentures can be repaired similarly to dentures manufactured by traditional methods, and it was confirmed that digitally manufactured dentures with aging surfaces can be repaired completely if appropriate surface treatment is performed [45]. Considering this, future studies should focus on dimensional stability, according to the storage environment of the 3D-printed denture base.

## 5. Conclusions

In denture base prostheses fabricated by 3D printing, the mandibular denture base had a larger dimensional change than the maxillary denture base after the postcuring process. Overall, long-term dimensional stability tended to increase with the postcuring time. The maxillary denture base had a similar dimensional change pattern regardless of the postcuring time, whereas the mandible had long term dimensional stability changes. Postcuring times of at least 30 min were found to increase long-term dimensional stability. The dimensional change according to the aging period was the largest between days 0 and 1, and the degree of change decreased as the period increased.

## Figures and Tables

**Table 1:** Specifications of the photopolymer resin.

Components	Amount
ethoxylated bisphenol A dimethacrylate	88–98%
2,4,6-Trimethylbenzoyl-diphenyl-diphenyl Phosphine oxide	2–5%

**Table 2:** Specifications of the 3D printer.

Printing Technology	LCD Type
Build volume	120 mm × 68 mm × 200 mm
Z-layer resolution	10 microns
XY resolution	47 microns
Light source	405-nm parallel aligned LED

**Table 3:** RMSE mean, standard deviation of trueness according to postcuring time and date after printing of the maxillary and mandibular denture bases. Unit of measure: µm.

	Minute	0 Days	1 Day	3 Days	7 Days	14 Days	28 Days
Maxilla	0	69.1 ± 13.3	105.5 ± 9.4	115.7 ± 3.4	127.8 ± 5.2	133.6 ± 4.9	131.2 ± 5.3
	5	57.7 ± 8.8	109.5 ± 8.9	119 ± 9.0	126.9 ± 9.9	135.6 ± 12.1	138.8 ± 12.6
	15	61.4 ± 3.7	108.8 ± 21.8	116.8 ± 31.2	125.2 ± 35.8	136.5 ± 44.6	144.7 ± 46.9
	30	61 ± 4.3	109.5 ± 35.1	120.2 ± 40.4	129.5 ± 46.7	130.9 ± 52.31	140.1 ± 54.1
	60	62.7 ± 8.0	107.5 ± 28.3	112.9 ± 34.4	115.7 ± 35.8	127.8 ± 40.3	126.6 ± 39.2
Mandible	0	104.2 ± 11.5	164.8 ± 14.9	182 ± 8.9	194.1 ± 10.7	199.3 ± 7.8	201.1 ± 5.5
	5	95.1 ± 3.0	186.2 ± 7.7	194 ± 9.3	204.3 ± 13.0	207.5 ± 12.8	206.6 ± 15.0
	15	95 ± 5.7	138.2 ± 10.2	155 ± 10.45	161.9 ± 8.8	166.7 ± 6.9	174.7 ± 7.0
	30	87.2 ± 6.12	116.6 ± 15.0	122.2 ± 17.6	129.4 ± 19.5	134 ± 18.9	131.5 ± 21.0
	60	88.5 ± 10.1	103.4 ± 13.6	110 ± 14.5	120.8 ± 15.4	129.3 ± 14.4	125.7 ± 13.0
